# Constraining the magnetohydrodynamic turbulence around Geminga by observing the γ-ray halo beyond 100 TeV

**DOI:** 10.1126/sciadv.adv8173

**Published:** 2026-03-04

**Authors:** The Tibet ASγ Collaboration

## Abstract

The extended γ-ray halo surrounding an old pulsar wind nebula (PWN) is an ideal place to investigate both the acceleration and diffusion processes of cosmic rays. In this work, we report the γ-ray halo observed with high precision by the Tibet ASγ experiment at the world’s highest-energy above 100 tera–electron volt (TeV). We determined the acceleration limit at about 100 TeV, beyond which the electron/positron flux suppresses exponentially, while we measured the morphology of the γ-ray halo at three different energies. We also found that the energy dependence of the diffusion coefficient is consistent with the Kolmogorov magnetohydrodynamic (MHD) turbulence and determined the turbulence property at scales smaller than 1 parsec. The measurements indicate that the energy of electrons/positrons is insufficient to amplify the strong MHD turbulence around itself, thereby suppressing the diffusion coefficient.

## Teaser

The Tibet ASγ experiment measured the subparsec-scale MHD turbulence in the Geminga γ-ray halo above 100 TeV.

## INTRODUCTION

Cosmic rays (CRs) are high-energy particles from space, mainly composed of protons and atomic nuclei with a small fraction of electrons, γ rays, and neutrinos, as well as fewer antiparticles (*1*). The antiparticles, such as antiprotons or positrons, are secondary particles produced by the interaction of CR protons/nuclei with the interstellar matter (ISM) during the CR propagation through the Galaxy (*2*). The charged protons and nuclei propagate diffusively in the Galaxy due to multiple interactions with the disordered magnetic field embedded in the interstellar medium (*3*, *4*). The diffusion picture describes propagation of CRs successfully. It explains most of the observed CR data on Earth, including the precise measurements of secondary particle spectra (*5*, *6*), except for a puzzling positron excess recently reported (*7*, *8*). It is found that the observed positron flux is much higher than that expected from the propagation model above about 10 GeV. This positron excess was measured by PAMELA (*7*) and confirmed by AMS-02 (*8*) with higher precision and up to higher energies.

It is generally believed that nearby pulsar wind nebulas (PWNs) are responsible for the extra high-energy positrons (*9*–*11*). A PWN contains a central rapidly spinning neutron star surrounded by extremely strong magnetic field, which blows out electron- and positron-winds interacting with the ISM and forming shock waves at the front (*12*). The injected electrons and positrons, which are further accelerated by the shock wave to very high energies, propagate diffusively to Earth and contribute to the local positron flux. However, a recent observation of TeV γ-ray halos around two nearby PWNs by the High-Altitude Water Cherenkov Observatory (HAWC) experiment made things more complicated. By analyzing the morphology of γ-ray halos, the HAWC experiment found that the electron/positron diffusion rate is too small to interpret the positron excess observed at Earth in terms of the contribution from nearby PWNs (*13*). Later, the High Energy Stereoscopic System (HESS) also measured the γ-ray spectrum from the halo center (*14*). Recently, HAWC updated the observation of the γ-ray halos with much higher precision (*15*).

How charged CRs propagate in the ISM is an essential aspect of astrophysics. A dominant interaction that CRs experience during their propagation is the pitch-angle scattering by the random magnetic field in the Galaxy (*4*). The interaction between CRs and magnetohydrodynamic (MHD) turbulence leads to diffusive propagation of CRs, which is characterized by the diffusion coefficient *D*. The widely accepted diffusion coefficient in the Galaxy is, however, constrained only globally based on the secondary nuclei abundance in CRs (*16*). The morphology of γ-ray halos around a point source, like a PWN, provides a unique measure of the local diffusion coefficient and, thereafter, the property of local MHD turbulence. The energy dependence of diffusion coefficient is especially useful for investigating the power spectrum of MHD turbulence, such as one predicted by the Kolmogorov theory (*17*). The Tibet ASγ experiment recently observed the γ-ray halo surrounding Geminga, a nearby pulsar at about 250 pc away from Earth (*18*). The morphology and energy spectrum of the Geminga γ-ray halo are measured from about 10 TeV up to beyond 100 TeV. Here, we derive the acceleration limit at the Geminga PWN. We also present the direct measurements of the CR diffusion coefficient as a function of energy and the property of MHD turbulence at tiny scales in the Galactic disk.

## RESULTS AND DISCUSSION

The electron/positron injection spectrum dominantly determines the energy spectrum of halo γ rays and is not very sensitive to the assumption of the energy dependence of diffusion coefficient *D*(*E*). Therefore, the electron injection spectrum is determined at first by assuming the diffusion coefficient expected from the Kolmogorov MHD turbulence, i.e., $D\left(E\right)\propto E^{1/3}$. We then get the electron spectrum by best-fitting this model to the observed radial distribution and energy spectrum of γ rays simultaneously.

**Fig. 1.**
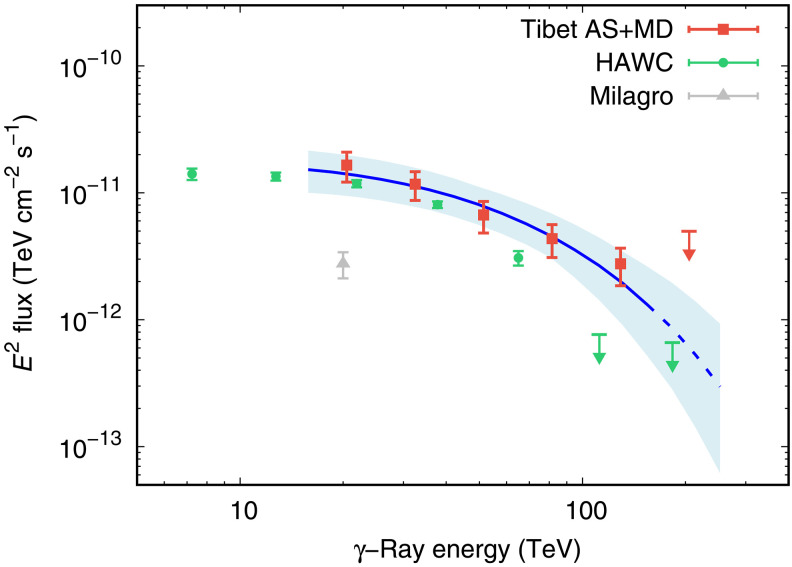
Energy spectrum of the Geminga halo γ rays measured by the Tibet ASγ experiment. The blue curve indicates the best-fit with the ECPL model, whereas the light blue band indicates the 1σ confidence interval of the fit. The spectrum is derived by fitting to the data below 158 TeV, whereas the red arrow at 251 TeV represents the flux upper limit at 90% C.L. The green points are the result by HAWC (*15*), and the gray point is the result by Milagro (*23*). It can be seen that the HAWC measurements (*15*) agree with this work below 50 TeV, whereas the Tibet ASγ experiment demonstrates superior sensitivity around 100 TeV. This is primarily due to its enhanced ability to reject high-energy CR backgrounds, enabled by a 2.5-m-deep underground MD array. Alternatively, there is a possibility of some systematic error between the Tibet ASγ and HAWC experiments.

The energy spectrum of halo γ rays measured by the Tibet ASγ experiment is very soft (see Fig. 1). It could be fitted by either a soft or hard power-law (PL) spectrum with a high-energy cutoff. We therefore perform best-fit calculations with two different forms of the injection spectrum: a PL and a PL with an exponential cutoff (ECPL), $q\left(E\right)=q_{0}{\left(E/1\text{TeV}\right)}^{-\alpha }\text{exp}\left(-E/E_{c}\right)$, where *E* is the energy of an electron or positron, α is the PL index, $E_{c}$ is the cutoff energy, and $q_{0}$ is the number of electrons/positrons at 1 TeV, far below $E_{c}$. We use the best-fit $q_{0}$ to estimate η, the conversion efficiency of pulsar’s spin-down energy dissipation to electron/positron energy. In the best-fitting with ECPL, the soft spectrum observed in a limited energy range results in low $E_{c}$, and the PL index α cannot be well constrained. In this case, therefore, we set α = 1 following the indication of x-ray measurements of the Geminga PWN (*19*–*21*).

We present the fitting results in Table 1. The PL model requires a soft spectrum with α ≈ 2.75, which results in a very large conversion efficiency η ≫ 1 and η < 1 is excluded at 3σ confidence level (C.L.). The PL model is strongly disfavored because η cannot exceed 1.0 unless there is an unknown energy source other than the Geminga pulsar itself, and thus the PL model is strongly disfavored. As for the ECPL model, on the other hand, the conversion efficiency is quite reasonable at η ∼ 0.1 (10%). The hard electron/positron spectrum with =1.0 below $E_{c}$ in the ECPL model is also supported by Fermi-LAT data (*22*), which set an upper limit on GeV γ-ray flux from the Geminga halo indicating a hard spectrum with α < 1.7. The cutoff energy in the ECPL model is $E_{c}∼100$ TeV, which gives the acceleration limit of the Geminga PWN. The Geminga PWN acceleration limit is determined from the γ-ray observation, thanks to the very high sensitivity of γ rays beyond 100 TeV of the Tibet ASγ experiment. As seen in Fig. 1, the γ-ray spectrum of the Geminga halo observed by the Tibet ASγ experiment is well fitted by the ECPL model. The HAWC measurement of the γ-ray spectrum (*15*) and the Milagro result (*23*) are also shown in the plot. It can be seen that the HAWC measurements (*15*) agree with this work below 50 TeV, whereas the Tibet ASγ experiment demonstrates superior sensitivity around 100 TeV. This enhanced performance stems from its superior background rejection capability for CRs at high energies, afforded by a 2.5-m-deep underground muon detector (MD) array. Alternatively, there is a possibility of some systematic error between the Tibet ASγ and HAWC experiments.

**Table 1.** Fitting results to the γ-ray spectrum and the SBPs of the Geminga halo. The PL model is disfavored as the energy conversion efficiency required by this model is higher than 100% at 3σ C.L. The cutoff energy representing the acceleration limit of the Geminga PWN is determined to be about 100 TeV. d.o.f., degrees of freedom.

	PL	ECPL
α	2.75 ± 0.16	–*
$E_{c}$ †	–	$98.0_{-19.5}^{+24.3}$
logη	2.48 ± 0.77	−0.94 ± 0.13
$D_{100}$ ‡	$1.37_{-0.47}^{+0.71}\times 10^{28}$	$1.26_{-0.42}^{+0.64}\times 10^{28}$
$\chi^{2}/\text{d.o.f.}$	0.73	0.84

* α is fixed to 1.0 in the ECPL model.

† In units of TeV.

‡ Diffusion coefficient at 100 TeV in units of cm^2^ s^−1^.

**Fig. 2.**
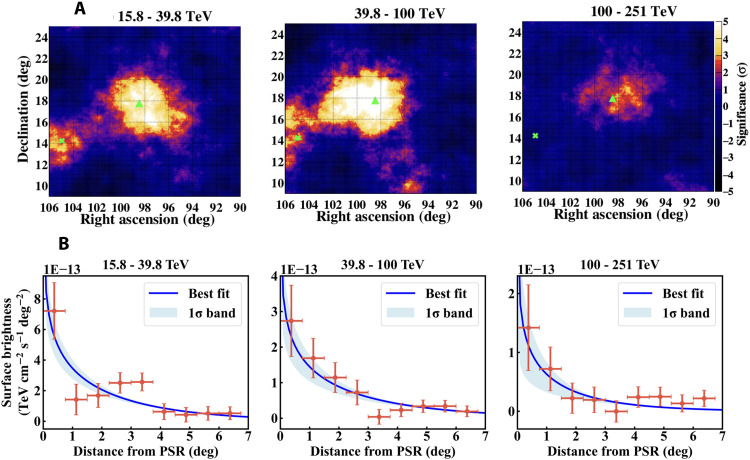
Two-dimensional significance maps of γ-ray emission from Geminga in three energy bins. (**A**) Each map is smoothed by a circular window with a radius of 3°. The green triangle in each map indicates the location of the Geminga pulsar, whereas the green X mark indicates the location of an another pulsar PSR B0656+14. (**B**) Data points display the observed radial profile of the surface brightness of halo γ rays, whereas blue curves show the best-fit results with the ECPL injection model in three energy bins. The diffusion coefficient in each energy bin is obtained individually. deg, degrees.

**Fig. 3.**
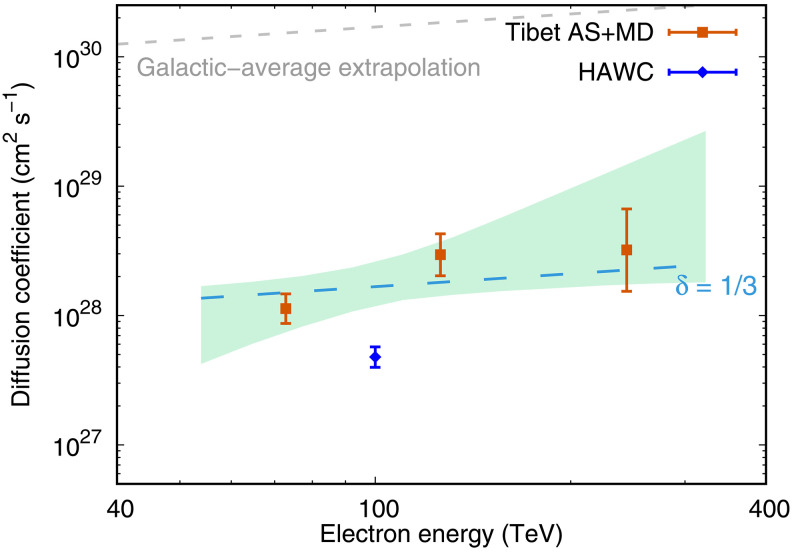
Energy dependence of the diffusion coefficient measured by the Tibet ASγ experiment. Red points display the diffusion coefficient obtained by fitting to the observed radial profile of halo γ rays in each of three energy bins shown in Fig. 2B. The electron energy of each point is calculated as the weighted average energy of electrons producing γ rays in each of three energy bins, i.e., 15.8 TeV < *E* < 39.8 TeV, 39.8 TeV < *E* < 100 TeV, and 100 TeV < *E* < 251 TeV, respectively. The green shaded area represents the 1σ confidence interval, evaluated by taking account of uncertainties in $D_{100}$ and δ. The light blue dashed line is the best-fit curve obtained by assuming δ = 1/3. The diffusion coefficient measured by HAWC (*15*) (blue point) is obviously lower than the result in this work, which may be due to differences in CR background estimation. The extrapolation from the average diffusion coefficient at the scale of whole Galaxy is also shown by the gray dashed line, which is derived from the boron/carbon ratio (*36*).

After getting electron/positron spectrum, we derive the diffusion coefficient *D*(*E*), from best-fit calculations to the radial profiles of halo γ rays in three energy bins, as shown in Fig. 2B. The best-fit *D*(*E*) at the three energy bins are shown in Fig. 3. The energy dependence of diffusion coefficient is determined by fitting a PL form of $D\left(E\right)=D_{100}{\left(E/100\;\text{TeV}\right)}^{\delta }$ with two free parameters, $D_{100}$ and δ. In Fig. 3, a 1σ confidence interval of the fit is indicated by the light green shaded area. The corresponding 1σ confidence interval of δ is 1.15 ± 0.55, whereas the possibility distribution of $D_{100}$ is integrated. The green dashed line in Fig. 3 is the best fit with δ = 1/3, which is expected from the Kolmogorov turbulence.

As seen in Fig. 3, the energy dependence of the diffusion coefficient obtained in this work is consistent with the expectation of Kolmogorov’s theory after considering the errors. This is the direct measurement of the energy dependence of the diffusion coefficient using the Tibet ASγ experimental data in an astrophysical environment. As actual tracers of high-energy electrons/positrons, the observed γ rays tell us how electrons/positrons diffuse away from the central PWN. It should be noted that the shaded region in Fig. 3 reflects the 68% confidence interval of the joint distribution of the two parameters, δ and $D_{100}$. It is usually wider than the marginal distribution for a single parameter δ.

**Fig. 4.**
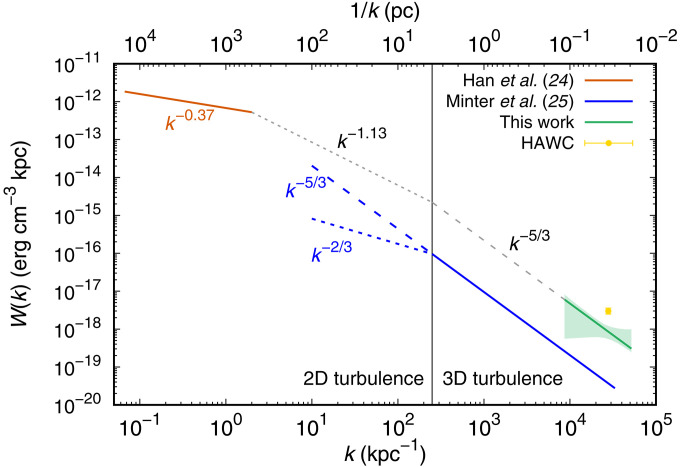
The power spectrum of the turbulent magnetic field in the interstellar medium. The power spectrum of magnetic turbulence measured in this work in comparison with those measured by Minter and Spangler (*25*) (blue solid line) and Han *et al.* (*24*) (red solid line). The yellow point is derived from the diffusion coefficient measured by HAWC (*15*). The turbulence is three-dimensional at a scale below ~4 pc, whereas it gradually turns to be two-dimensional above ~4 pc. For three-dimensional turbulence, the spectral index of −5/3 is expected from the Kolmogorov theory.

Furthermore, we derive from the obtained *D*(*E*) the power spectrum of MHD turbulence, which is closely related to *D*(*E*) as expected from the resonant pitch angle scattering of electrons/protons by the turbulence. In Fig. 4, we summarize the magnetic turbulence power spectra derived by different methods. Han *et al.* (*24*) obtained an average power spectrum over one-third of the Galactic disk by using the rotation measure and dispersion measure of Galactic pulsars. Minter and Spangler (*25*), on the other hand, derived the magnetic turbulence over a galactic anticenter sky including the Galactic halo. In Fig. 4, the theoretical extrapolation of the result by Han *et al.* (*24*) to a smaller scale is compared with the result derived in the present work. The power spectrum obtained in the present work below the ~4-pc scale is extrapolated with the spectral index of −5/3 expected from Kolmogorov turbulence (black dashed line). The spectral index above the ~4-pc scale is uncertain as the turbulence gradually turns to be two-dimensional with the spectral index lying between −5/3 and −2/3 (black dotted line) (*25*). As seen in Fig. 4, the spectrum derived in this work is well consistent with the theoretical extrapolation from the spectrum by Han *et al.* (*24*). It is evident in this figure, on the other hand, that the magnetic turbulence power measured by Minter and Spangler (*25*) in the Galactic halo is much weaker than the average values by Han *et al.* (*24*) at the Galactic disk scale and that by the present work at a much smaller scale. The amplitude of magnetic turbulence in the Galactic disk may be much larger than in the Galactic halo. Figure 4 also implies that the MHD turbulence environment around Geminga may be common in the Galactic disk. We reveal the power spectrum of MHD turbulence at such a small scale in the Galactic disk.

We lastly add that the present result has another important implication about the origin of strong MHD turbulence and small diffusion coefficient. A straightforward explanation of such a strong turbulence might be that electrons and positrons accelerated and released from the PWN stimulate MHD turbulence in the region around the PWN (*26*, *27*). Large flux of electron-positron pairs in the vicinity of the PWN may amplify the resonant magnetic waves, and the amplified waves may, in turn, confine CR particles around the PWN. As seen in Fig. 1, however, the electron/positron flux above 100 TeV is obviously suppressed whereas the diffusion coefficient at *E* > 100 TeV is consistent with the behavior of Kolmogorov-type turbulence. We calculated the energy density of magnetic turbulence at the highest energy bin and found that the electron/positron energy density in this energy bin is about two orders of magnitude smaller than the magnetic turbulence energy. Therefore, external energy supplies are needed to produce enough turbulence to suppress diffusion. A possible energy supply is expected from the shock wave in its progenitor supernova remnant, as suggested by Fang *et al.* (*28*).

On the basis of the above results, the Tibet ASγ experiment has made a beautiful measurement of a γ-ray halo surrounding the Geminga PWN in an energy region up to the highest energy above 100 TeV. The energy spectrum of electrons/positrons injected from the PWN and the diffusion coefficient at different energies are determined simultaneously. The acceleration limit for the Geminga PWN is determined in this work. This measurement also indicates that MHD turbulence in the region around the Geminga PWN is consistent with that of Kolmogorov type. This work presents the direct measurement of the CR diffusion coefficient as a function of energy and the property of MHD turbulence at tiny scales in the Galactic disk. The turbulence property obtained in this work is consistent with the extrapolation from measurements of the Galactic disk turbulence at larger scales. The Galactic disk seems to have a stronger magnetic turbulence than that in the Galactic halo. The present work also shows that the origin of strong turbulence around the Geminga PWN might be an environmental effect.

## MATERIALS AND METHODS

### Experiment

The Tibet air shower (AS) array has operated since 1990 in Yangbajing (90.522°E, 30.102°N; 4300 m above sea level) in Tibet, China and observing CRs and γ rays above TeV energies. The Tibet ASγ experiment consists of a surface AS array with an underground MD array (*29*). We equip the AS array with 597 plastic scintillation detectors, each covering 0.5 m^2^, achieving a total detection area of 65,700 m^2^. We design the MD array to span 3400 m^2^, using water-Cherenkov detectors to capture Cherenkov light from shower muons passing through the water layer. We construct each MD detector with water pools partitioned by concrete and bury it 2.4 m underground. The combined soil overburden and concrete shielding equivalent to ~19 radiation lengths, which restricts access to the water layer to only those muons with energies exceeding 1 GeV (for vertical incidence). By using the MD array, we enhance the experiment’s sensitivity to 100-TeV gamma rays by over an order of magnitude, by rejecting more than 99.9% of hadronic CRs while keeping 90% of gamma rays around the 100-TeV energy range (*29*, *30*). The angular resolutions of arrival direction (50% containment) is estimated to be ~0.5° and 0.2° for 10- and 100-TeV primary γ rays, respectively (*29*). We reconstruct the primary particle’s energy using measured particle densities from AS detectors. For energies above 10 TeV, we fit the lateral density distribution with the Nishimura-Kamata-Greisen (NKG) function and derive the particle density at 50 m from the shower axis (S50). We then determine the reconstructed gamma-ray energy (Erec) as a function of S50 and the zenith angle. We estimate the energy resolution to be about 40% at 10 TeV, improving to 20% at 100 TeV for primary gamma rays, respectively (*31*).

In this work, we use the data collected by the Tibet AS array combined with the underground MD array during 719 live days between February 2014 and May 2017. Our data selection criteria are the same as those described in our previous paper (*29*). For more details including our muon-cut threshold, please see our previous paper (*29*).

### Data analysis

To estimate the background contribution from CRs when analyzing γ-ray emission from Geminga pulsar ($\alpha =06^{h}33^{m}54.153^{s}$, $\delta =+17^{\circ }46^{\prime }12.9{1^{\prime }}^{\prime }$), we use the “equi-declination method” in which an event observed at the same declination as Geminga but at a different right ascension is regarded as a background event. In a declination band including Geminga, we first set adjacent 11 circular windows, each with an angular radius of 10°, and define the middle window centered at Geminga as the “ON-source” window and the remaining 10 windows on both sides of Geminga as the “OFF-source” windows. We then divide each window into the inner window with 3° radius and the outer ring (the outer window) outside the inner window. In this method, we assume that the background contribution in the inner “ON-source” window is the same as that in the outer “ON-source” window. We estimate the anisotropy contribution to the outer “ON-source” window by $k={\bar{N}}_{\mathrm{BG}}^{\text{out}}/N_{\mathrm{ON}}^{\text{out}}$, where $N_{\mathrm{ON}}^{\text{out}}$ is the event number in the outer “ON-source” window and ${\bar{N}}_{\mathrm{BG}}^{\text{out}}$ is the average background event number in outer “OFF-source” windows given by ${\bar{N}}_{\mathrm{BG}}^{\text{out}}=\sum_{i=1}^{10}N_{\mathrm{OFF},i}^{\text{out}}/10$ with $N_{\mathrm{OFF},i}^{\text{out}}$ denoting the event number in the *i*th outer “OFF-source” window. By using this *k*, we calculate the signal event number $N_{S}$ in the inner “ON-source” window as $N_{S}=N_{\mathrm{ON}}^{\text{in}}-k{\bar{N}}_{\mathrm{BG}}^{\text{in}}$ where $N_{\mathrm{ON}}^{\text{in}}$ is the event number in the inner “ON-source” window and ${\bar{N}}_{\mathrm{BG}}^{\text{in}}$ is the average background event number in inner “OFF-source” windows given by ${\bar{N}}_{\mathrm{BG}}^{\text{in}}=\sum_{i=1}^{10}N_{\mathrm{OFF},i}^{\text{in}}/10$ with $N_{\mathrm{OFF},i}^{\text{in}}$ denoting the event number in the *i*th inner “OFF-source” window.

We divide datasets into the following three energy bins above 10 TeV. Figure 2A shows the significance map of the observed γ-ray emission around the Geminga pulsar in three energy bins, each as a function of the declination and right ascension. Each map is smoothed with a circular window with an angular radius of 3°. In 15.8 to 39.8 TeV, γ-ray emission is seen around the Geminga pulsar with 4.7σ Li-Ma significance. The total $N_{\text{ON}}$ and $N_{\text{BG}}$ in this energy bin are 12,721 and 12,177, respectively. We see a separate ~3σ excess around the PSR B0656+14 (Monogem) (*32*–*34*). As energy increases, in 39.8 to 100 TeV, the average significance of γ rays from Geminga increases to 5.3σ with $N_{\text{ON}}$ = 1594 and $N_{\text{BG}}$ = 1380. As energy increases further, in 100 to 251 TeV, the average significance decreases to 2.3σ with $N_{\text{ON}}$ = 168 and $N_{\text{BG}}$ = 138 due to a rapid decrease in event number. The combined significance of the Geminga halo reaches 6.3σ.

### Model construction

Electrons and positrons are accelerated by the PWN and injected into the ISM surrounding the PWN. These particles propagate in the ISM diffusively and produce the γ-ray halo through inverse Compton scattering (ICS) with the background radiation. By solving the diffusive propagation equation (eq. S1 in the Supplementary Materials), we get the current electron/positron density distribution N(E,r) in the halo, where *E* and ***r*** are the electron/positron energy and position, respectively. Then, we obtain the electron/positron surface density $S_{e}\left(E,\theta \right)$ at an angular distance θ from Geminga by integrating N(E,r) along the line of sight as

$$S_{e}\left(E,\theta \right)=\int_{0}^{\infty }N\left(E,r\right)\mathrm{dl}$$ (1)

where $|r|=\sqrt{d^{2}+l^{2}-2\mathrm{dl}\text{cos}\theta }$, and *d* is the distance between Geminga and Earth. Subsequently, the energy-differential surface brightness profile (SBP) around Geminga can be computed by

$$s_{\gamma }\left(E_{\gamma },\theta \right)=\iint S_{e}\left(E,\theta \right)f\left(E,E_{\gamma },\nu \right)\text{dνdE}$$ (2)

where $E_{\gamma }$ is energy of emitted γ rays, ν is the background photon energy, and $f\left(E,E_{\gamma },\nu \right)$ is the emitted γ-ray energy distribution for incident electrons/positrons with energy *E* and target photons with energy ν (*35*).

The energy-integrated SBP in a given energy range of $\left(E_{\gamma ,1},E_{\gamma ,2}\right)$, as shown in Fig. 2, is calculated by

$$S_{\gamma }\left(\theta \right)=\int_{E_{\gamma ,1}}^{E_{\gamma ,2}}s_{\gamma }\left(E_{\gamma },\theta \right)E_{\gamma }{\mathrm{dE}}_{\gamma }$$ (3)

The γ-ray SBP reflects the spatial distribution of electrons/positrons and is sensitive to the diffusion coefficient, indicating that the diffusion coefficient can be determined by fitting the SBP to the data.

The total γ-ray spectrum of the halo, as shown in Fig. 1, is calculated by

$$F\left(E_{\gamma }\right)=\int_{0}^{\infty }s_{\gamma }\left(E_{\gamma },\theta \right)2\pi \theta d\theta$$ (4)

The γ-ray spectrum is predominantly determined by the injection energy spectrum of electrons/positrons. This implies that the spectral data can be used to derive the parameters of the injection spectrum.

## Supplementary Materials

### This PDF file includes:

The Tibet ASγ Collaboration Author list Supplementary Text Fig. S1 References

## References

[1] P. Blasi, The origin of galactic cosmic rays. Astron. Astrophys. Rev. 21, 70 (2013).

[2] T. K. Gaisser, *Cosmic Rays and Particle Physics* (Cambridge Univ. Press, 1990).

[3] V. L. Ginzburg, Cosmic rays and plasma phenomena in the Galaxy and Metagalaxy. Sov. Astron. 9, 877 (1966).

[4] V. S. Berezinsky, S. V. Bulanov, V. A. Dogiel, V. L. Ginzburg, V. S. Ptuskin, *Astrophysics of Cosmic Rays* (North-Holland, 1990).

[5] O. Adriani, G. C. Barbarino, G. A. Bazilevskaya, R. Bellotti, M. Boezio, E. A. Bogomolov, L. Bonechi, M. Bongi, V. Bonvicini, S. Borisov, S. Bottai, A. Bruno, F. Cafagna, D. Campana, R. Carbone, P. Carlson, M. Casolino, G. Castellini, L. Consiglio, M. P. De Pascale, C. De Santis, N. De Simone, V. Di Felice, A. M. Galper, W. Gillard, L. Grishantseva, P. Hofverberg, G. Jerse, A. V. Karelin, S. V. Koldashov, S. Y. Krutkov, A. N. Kvashnin, A. Leonov, V. Malvezzi, L. Marcelli, A. G. Mayorov, W. Menn, V. V. Mikhailov, E. Mocchiutti, A. Monaco, N. Mori, N. Nikonov, G. Osteria, P. Papini, M. Pearce, P. Picozza, C. Pizzolotto, M. Ricci, S. B. Ricciarini, L. Rossetto, M. Simon, R. Sparvoli, P. Spillantini, Y. I. Stozhkov, A. Vacchi, E. Vannuccini, G. Vasilyev, S. A. Voronov, J. Wu, Y. T. Yurkin, G. Zampa, N. Zampa, V. G. Zverev, PAMELA results on the cosmic-ray antiproton flux from 60 MeV to 180 GeV in kinetic energy. Phys. Rev. Lett. 105, 121101 (2010). 20867623 10.1103/PhysRevLett.105.121101

[6] M. Aguilar, L. A. Cavasonza, B. Alpat, G. Ambrosi, L. Arruda, N. Attig, S. Aupetit, P. Azzarello, AMS Collaboration, Antiproton flux, antiproton-to-proton flux ratio, and properties of elementary particle fluxes in primary cosmic rays measured with the Alpha Magnetic Spectrometer on the International Space Station. Phys. Rev. Lett. 117, 091103 (2016). 27610839 10.1103/PhysRevLett.117.091103

[7] O. Adriani, G. C. Barbarino, G. A. Bazilevskaya, R. Bellotti, M. Boezio, E. A. Bogomolov, L. Bonechi, M. Bongi, V. Bonvicini, S. Bottai, A. Bruno, F. Cafagna, D. Campana, R. Carbone, M. Casolino, G. Castellini, M. P. De Pascale, G. De Rosa, C. De Santis, N. De Simone, V. Di Felice, A. M. Galper, L. Grishantseva, P. Hofverberg, S. V. Koldashov, S. Y. Krutkov, A. N. Kvashnin, A. Leonov, V. Malvezzi, L. Marcelli, W. Menn, V. V. Mikhailov, E. Mocchiutti, S. Orsi, G. Osteria, P. Papini, M. Pearce, P. Picozza, M. Ricci, S. B. Ricciarini, M. Simon, R. Sparvoli, P. Spillantini, Y. I. Stozhkov, A. Vacchi, E. Vannuccini, G. Vasilyev, S. A. Voronov, Y. T. Yurkin, G. Zampa, N. Zampa, V. G. Zverev, An anomalous positron abundance in cosmic rays with energies 1.5–100 GeV. Nature 458, 607–609 (2009). 19340076 10.1038/nature07942

[8] L. Accardo, M. Aguilar, D. Aisa, B. Alpat, A. Alvino, G. Ambrosi, K. Andeen, L. Arruda, AMS Collaboration, High statistics measurement of the positron fraction in primary cosmic rays of 0.5-500 GeV with the alpha magnetic spectrometer on the international space station. Phys. Rev. Lett. 113, 121101 (2014). 25279616 10.1103/PhysRevLett.113.121101

[9] D. Hooper, P. Blasi, P. D. Serpico, Pulsars as the sources of high energy cosmic ray positrons. J. Cosmol. Astropart. Phys. 2009, 025 (2009).

[10] P. F. Yin, Z. H. Yu, Q. Yuan, X. J. Bi, Pulsar interpretation for the AMS-02 result. Phys. Rev. D 88, 023001 (2013).

[11] S. Profumo, Dissecting cosmic-ray electron-positron data with Occam’s Razor: The role of known Pulsars. Central Eur. J. Phys. 10, 1–31 (2012).

[12] B. M. Gaensler, P. O. Slane, The evolution and structure of pulsar wind nebulae. Ann. Rev. Astron. Astrophys. 44, 17–47 (2006).

[13] A. U. Abeysekara, A. Albert, R. Alfaro, C. Alvarez, J. D. Álvarez, R. Arceo, J. C. Arteaga-Velázquez, D. Avila Rojas, H. A. Ayala Solares, A. S. Barber, N. Bautista-Elivar, A. Becerril, E. Belmont-Moreno, S. Y. BenZvi, D. Berley, A. Bernal, J. Braun, C. Brisbois, K. S. Caballero-Mora, T. Capistrán, A. Carramiñana, S. Casanova, M. Castillo, U. Cotti, J. Cotzomi, S. Coutiño de León, C. De León, E. De la Fuente, B. L. Dingus, M. A. DuVernois, J. C. Díaz-Vélez, R. W. Ellsworth, K. Engel, O. Enríquez-Rivera, D. W. Fiorino, N. Fraija, J. A. García-González, F. Garfias, M. Gerhardt, A. González Muñoz, M. M. González, J. A. Goodman, Z. Hampel-Arias, J. P. Harding, S. Hernández, A. Hernández-Almada, J. Hinton, B. Hona, C. M. Hui, P. Hüntemeyer, A. Iriarte, A. Jardin-Blicq, V. Joshi, S. Kaufmann, D. Kieda, A. Lara, R. J. Lauer, W. H. Lee, D. Lennarz, H. León Vargas, J. T. Linnemann, A. L. Longinotti, G. Luis Raya, R. Luna-García, R. López-Coto, K. Malone, S. S. Marinelli, O. Martinez, I. Martinez-Castellanos, J. Martínez-Castro, H. Martínez-Huerta, J. A. Matthews, P. Miranda-Romagnoli, E. Moreno, M. Mostafá, L. Nellen, M. Newbold, M. U. Nisa, R. Noriega-Papaqui, R. Pelayo, J. Pretz, E. G. Pérez-Pérez, Z. Ren, C. D. Rho, C. Rivière, D. Rosa-González, M. Rosenberg, E. Ruiz-Velasco, H. Salazar, F. Salesa Greus, A. Sandoval, M. Schneider, H. Schoorlemmer, G. Sinnis, A. J. Smith, R. W. Springer, P. Surajbali, I. Taboada, O. Tibolla, K. Tollefson, I. Torres, T. N. Ukwatta, G. Vianello, T. Weisgarber, S. Westerhoff, I. G. Wisher, J. Wood, T. Yapici, G. Yodh, P. W. Younk, A. Zepeda, H. Zhou, F. Guo, J. Hahn, H. Li, H. Zhang, Extended gamma-ray sources around pulsars constrain the origin of the positron flux at Earth. Science 358, 911 (2017). 29146808 10.1126/science.aan4880

[14] A. M. W. Mitchell, S. Caroff, J. Hintonc, L. Mohrmannd on behalf of the H.E.S.S. Collaboration, Detection of extended TeV emission around the Geminga pulsar with H.E.S.S. arXiv:2108.02556 [astro-ph.HE] (2022).

[15] A. Albert, R. Alfaro, C. Alvarez, J. C. Arteaga-Velázquez, D. Avila Rojas, H. A. Ayala Solares, R. Babu, E. Belmont-Moreno, A. Bernal, K. S. Caballero-Mora, T. Capistrán, A. Carramiñana, S. Casanova, U. Cotti, J. Cotzomi, S. Coutiño de León, E. de la Fuente, D. Depaoli, N. Di Lalla, R. Diaz Hernandez, B. L. Dingus, M. A. DuVernois, M. Durocher, J. C. Díaz-Vélez, K. Engel, C. Espinoza, K. L. Fan, K. Fang, N. Fraija, J. A. García-González, F. Garfias, H. Goksu, M. M. González, J. A. Goodman, S. Groetsch, J. P. Harding, S. Hernández-Cadena, I. Herzog, P. Hüntemeyer, D. Huang, F. Hueyotl-Zahuantitla, A. Iriarte, V. Joshi, S. Kaufmann, D. Kieda, A. Lara, W. H. Lee, J. Lee, H. León Vargas, J. T. Linnemann, A. L. Longinotti, G. Luis-Raya, K. Malone, O. Martinez, J. Martínez-Castro, J. A. Matthews, P. Miranda-Romagnoli, J. A. Montes, J. A. Morales-Soto, E. Moreno, M. Mostafá, A. Nayerhoda, L. Nellen, R. Noriega-Papaqui, L. Olivera-Nieto, N. Omodei, Y. Pérez Araujo, E. G. Pérez-Pérez, C. D. Rho, D. Rosa-González, H. Salazar, D. Salazar-Gallegos, A. Sandoval, M. Schneider, G. Schwefer, J. Serna-Franco, Y. Son, R. W. Springer, O. Tibolla, K. Tollefson, I. Torres, R. Torres-Escobedo, R. Turner, F. Urea-Mena, E. Varela, L. Villaseñor, X. Wang, I. J. Watson, E. Willox, H. Wu, S. Yun-Cárcamo, H. Zhou, C. de León, M. Di Mauro, Precise measurements of TeV halos around Geminga and Monogem pulsars with HAWC. Astrophys. J. 974, 246–258 (2024).

[16] M. Aguilar, L. A. Cavasonza, G. Ambrosi, L. Arruda, N. Attig, S. Aupetit, P. Azzarello, A. Bachlechner, AMS Collaboration, Precision measurement of the boron to carbon flux ratio in cosmic rays from 1.9 GV to 2.6 TV with the Alpha Magnetic Spectrometer on the International Space Station. Phys. Rev. Lett. 117, 231102 (2016). 27982618 10.1103/PhysRevLett.117.231102

[17] A. Kolmogorov, The local structure of turbulence in incompressible viscous fluid for very large Reynolds numbers. Dokl. Akad. Nauk SSSR 30, 301–305 (1941).

[18] J. Faherty, F. M. Walter, J. Anderson, The trigonometric parallax of the neutron star Geminga. Astrophys. Space Sci. 308, 225–230 (2007).

[19] G. G. Pavlov, S. Bhattacharyya, V. E. Zavlin, New x-ray observations of the Geminga pulsar wind nebula. Astrophys. J. 715, 66–77 (2010).

[20] B. Posselt, G. G. Pavlov, P. O. Slane, R. Romani, N. Bucciantini, A. M. Bykov, O. Kargaltsev, M. C. Weisskopf, C. Y. Ng, Geminga’s puzzling pulsar wind nebula. Astrophys. J. 835, 66 (2017).

[21] P. A. Caraveo, G. F. Bignami, A. DeLuca, S. Mereghetti, A. Pellizzoni, R. Mignani, A. Tur, W. Becker, Geminga’s tails: A pulsar bow shock probing the interstellar medium. Science 301, 1345–1347 (2003). 12881574 10.1126/science.1086973

[22] S.-Q. Xi, R.-Y. Liu, Z.-Q. Huang, K. Fang, X.-Y. Wang, GeV observations of the extended pulsar wind nebulae constrain the pulsar interpretations of the cosmic-ray positron excess. Astrophys. J. 878, 104 (2019).

[23] A. A. Abdo, B. T. Allen, T. Aune, D. Berley, C. Chen, G. E. Christopher, T. DeYoung, B. L. Dingus, R. W. Ellsworth, M. M. Gonzalez, J. A. Goodman, E. Hays, C. M. Hoffman, P. H. Huntemeyer, B. E. Kolterman, J. T. Linnemann, J. E. McEnery, T. Morgan, A. I. Mincer, P. Nemethy, J. Pretz, J. M. Ryan, P. M. S. Parkinson, A. Shoup, G. Sinnis, A. J. Smith, V. Vasileiou, G. P. Walker, D. A. Williams, G. B. Yodh, Milagro observations of multi-TeV emission from galactic sources in the fermi bright source list. Astrophys. J. Lett. 700, L127–L131 (2009).

[24] J. L. Han, K. Ferriere, R. N. Manchester, The spatial energy spectrum of magnetic fields in our galaxy. Astrophys. J. 610, 820–826 (2004).

[25] A. H. Minter, S. R. Spangler, Observation of turbulent fluctuations in the interstellar plasma density and magnetic field on spatial scales of 0.01 to 100 parsecs. Astrophys. J. 458, 194 (1996).

[26] C. Evoli, T. Linden, G. Morlino, Self-generated cosmic-ray confinement in TeV halos: Implications for TeV γ-ray emission and the positron excess. Phys. Rev. D 98, 063017 (2018).

[27] P. Mukhopadhyay, T. Linden, Self-generated cosmic-ray turbulence can explain the morphology of TeV halos. Phys. Rev. D 105, 123008 (2022).

[28] K. Fang, X.-J. Bi, P.-F. Yin, Possible origin of the slow-diffusion region around Geminga. Mon. Not. R. Astron. Soc. 488, 4074–4080 (2019).

[29] M. Amenomori, Y. W. Bao, X. J. Bi, D. Chen, T. L. Chen, W. Y. Chen, X. Chen, Y. Chen, Tibet ASγ Collaboration, First detection of photons with energy beyond 100 TeV from an astrophysical source. Phys. Rev. Lett. 123, 051101 (2019). 31491288 10.1103/PhysRevLett.123.051101

[30] T. K. Sako, K. Kawata, M. Ohnishi, A. Shiomi, M. Takita, H. Tsuchiya, Exploration of a 100 TeV gamma-ray northern sky using the Tibet air-shower array combined with an underground water-Cherenkov muon-detector array. Astropart. Phys. 32, 177–184 (2009).

[31] K. Kawata, T. K. Sako, M. Ohnishi, M. Takita, Y. Nakamura, K. Munakata, Energy determination of gamma-ray induced air showers observed by an extensive air shower array. Exp. Astron. 44, 1–9 (2017).

[32] M. Amenomori, X. J. Bi, D. Chen, S. W. Cui, Danzengluobu, L. K. Ding, X. H. Ding, C. Fan, C. F. Feng, Z. Feng, Z. Y. Feng, X. Y. Gao, Q. X. Geng, H. W. Guo, H. H. He, M. He, K. Hibino, N. Hotta, H. Hu, H. B. Hu, J. Huang, Q. Huang, H. Y. Jia, F. Kajino, K. Kasahara, Y. Katayose, C. Kato, K. Kawata, Labaciren, G. M. Le, A. F. Li, J. Y. Li, Y.-Q. Lou, H. Lu, S. L. Lu, X. R. Meng, K. Mizutani, J. Mu, K. Munakata, A. Nagai, H. Nanjo, M. Nishizawa, M. Ohnishi, I. Ohta, H. Onuma, T. Ouchi, S. Ozawa, J. R. Ren, T. Saito, T. Y. Saito, M. Sakata, T. K. Sako, M. Shibata, A. Shiomi, T. Shirai, H. Sugimoto, M. Takita, Y. H. Tan, N. Tateyama, S. Torii, H. Tsuchiya, S. Udo, B. Wang, H. Wang, X. Wang, Y. Wang, Y. G. Wang, H. R. Wu, L. Xue, Y. Yamamoto, C. T. Yan, X. C. Yang, S. Yasue, Z. H. Ye, G. C. Yu, A. F. Yuan, T. Yuda, H. M. Zhang, J. L. Zhang, N. J. Zhang, X. Y. Zhang, Y. Zhang, Y. Zhang, Zhaxisangzhu, X. X. Zhou, Multi-TeV gamma-ray observation from the crab nebula using the Tibet-III air shower array finely tuned by the cosmic ray Moon’s shadow. Astrophys. J. 692, 61–72 (2009).

[33] A. A. Abdo, B. Allen, T. Aune, D. Berley, E. Blaufuss, S. Casanova, C. Chen, B. L. Dingus, R. W. Ellsworth, L. Fleysher, R. Fleysher, M. M. Gonzalez, J. A. Goodman, C. M. Hoffman, P. H. Huntemeyer, B. E. Kolterman, C. P. Lansdell, J. T. Linnemann, J. E. McEnery, A. I. Mincer, P. Nemethy, D. Noyes, J. Pretz, J. M. Ryan, P. M. Saz Parkinson, A. Shoup, G. Sinnis, A. J. Smith, G. W. Sullivan, V. Vasileiou, G. P. Walker, D. A. Williams, G. B. Yodh, Discovery of localized regions of excess 10-TeV cosmic rays. Phys. Rev. Lett. 101, 221101 (2008). 19113471 10.1103/PhysRevLett.101.221101

[34] M. Amenomori, S. Ayabe, S. W. Cui, Danzengluobu, L. K. Ding, X. H. Ding, C. F. Feng, Z. Y. Feng, X. Y. Gao, Q. X. Geng, H. W. Guo, H. H. He, M. He, K. Hibino, N. Hotta, H. Hu, H. B. Hu, J. Huang, Q. Huang, H. Y. Jia, F. Kajino, K. Kasahara, Y. Katayose, K. Kawata, Labaciren, G. M. Le, J. Y. Li, H. Lu, S. L. Lu, X. R. Meng, K. Mizutani, J. Mu, H. Nanjo, M. Nishizawa, M. Ohnishi, I. Ohta, T. Ouchi, S. Ozawa, J. R. Ren, T. Saito, M. Sakata, T. Sasaki, M. Shibata, A. Shiomi, T. Shirai, H. Sugimoto, K. Taira, M. Takita, Y. H. Tan, N. Tateyama, S. Torii, H. Tsuchiya, S. Udo, T. Utsugi, B. S. Wang, H. Wang, X. Wang, Y. G. Wang, L. Xue, Y. Yamamoto, X. C. Yang, Z. H. Ye, G. C. Yu, A. F. Yuan, T. Yuda, H. M. Zhang, J. L. Zhang, N. J. Zhang, X. Y. Zhang, Y. Zhang, Zhaxisangzhu, X. X. Zhou, Multi-tev gamma-ray flares from markarian 421 in 2000 and 2001 observed with the Tibet air shower array. Astrophys. J. 598, 242–249 (2003).

[35] G. R. Blumenthal, R. J. Gould, Bremsstrahlung, synchrotron radiation, and Compton scattering of high-energy electrons traversing dilute gases. Rev. Mod. Phys. 42, 237–270 (1970).

[36] Q. Yuan, S.-J. Lin, K. Fang, X.-J. Bi, Propagation of cosmic rays in the AMS-02 era. Phys. Rev. D 95, 083007 (2017).

[37] K. Fang, X. J. Bi, S. J. Lin, Q. Yuan, Klein-Nishina effect and the cosmic ray electron spectrum. Chin. Phys. Lett. 38, 039801 (2021).

[38] T. Delahaye, J. Lavalle, R. Lineros, F. Donato, N. Fornengo, Galactic electrons and positrons at the Earth: New estimate of the primary and secondary fluxes. Astron. Astrophys. 524, A51 (2010).

[39] F. Feroz, M. P. Hobson, M. Bridges, MultiNest: An efficient and robust Bayesian inference tool for cosmology and particle physics. Mon. Not. R. Astron. Soc. 398, 1601–1614 (2009).

[40] A. Lewis, GetDist: A Python package for analysing Monte Carlo samples. arXiv:1910.13970 [astro-ph.IM] (2019).

