# Supplementary material for: Constraining the magnetohydrodynamic turbulence around Geminga by observing the γ-ray halo beyond 100 TeV
Source: Sci Adv. 2026 Mar 4;12(10):eadv8173. doi: 10.1126/sciadv.adv8173 (PMC12959393; doi:10.1126/sciadv.adv8173)
Supplement: Supplementary file 1 — The Tibet ASγ Collaboration Author list Supplementary Text Fig. S1 References [file sciadv.adv8173_sm.pdf]

Science Advances
AAAS

Supplementary Materials for

Constraining the magnetohydrodynamic turbulence around Geminga by observing the γ-ray halo beyond 100 TeV

The Tibet ASγ Collaboration

Corresponding author: Jing Huang, huangjing@ihep.ac.cn; Masato Takita, takita@icrr.u-tokyo.ac.jp; Xiaojun Bi, bixj@ihep.ac.cn; Ding Chen, chending@nao.cas.cn; Kun Fang, fangkun@ihep.ac.cn; Yusaku Katayose, katayose-yusaku-dv@ynu.ac.jp; Kazumasa Kawata, kawata@icrr.u-tokyo.ac.jp; Munehiro Ohnishi, ohnishi@icrr.u-tokyo.ac.jp; Atsushi Shiomi, shiomi.atsushi@nihon-u.ac.jp

Sci. Adv. **12**, eadv8173 (2026)
DOI: 10.1126/sciadv.adv8173

This PDF file includes:

The Tibet ASγ Collaboration Author list
Supplementary Text
Fig. S1
References

The Tibet ASγ Collaboration

Michihiro Amenomori[1], Marcos Anzorena[2], Yiwei Bao[3], Xiaojun Bi[4*], Ding Chen[5*], Tianlu Chen[6], Yang Chen[3], Cirennima[6], Shuwang Cui[7], Danzengluobu[6], Jianhua Fang[8], Kun Fang[4*], Cunfeng Feng[9], Youliang Feng[6], Qi Gao[6], Quanbu Gou[4], Rocio Garcia[2], Chenyao Han[10], Yuki Hayashi[11], Huihai He[4], Kinya Hibino[12], Naoki Hotta[13], Haibing Hu[6], Kongyi Hu[4,14], Jing Huang[4*], Ginga Imaizumi[2], Huanyu Jia[15], Katsuaki Kasahara[16], Yusaku Katayose[17*], Chihiro Kato[11], Sei Kato[18], Teruyoshi Kawashima[2], Kazumasa Kawata[2*], Masayoshi Kozai[19], Labaciren[6], Guiming Le[20], Haijin Li[6], YiYang Li[4,14], Yuhui Lin[21], Liyong Liu[5], Maoyuan Liu[6], Hong Lu[4], Yu Meng[4,14], Atsushi Mizuno[2], Kazuoki Munakata[11], Changchun Ning[6], Masaki Nishizawa[22], Munehiro Ohnishi[2*], Sosuke Okukawa[17], Xiangli Qian[23], Xiaobo Qu[24], Toshiharu Saito[25], Takashi Sako[2], Takashi K. Sako[26], Quanqi Shi[10], Makio Shibata[17], Atsushi Shiomi[27*], Futatsu Sugimoto[2], Wakiko Takano[12], Masato Takita[2*], Youheng Tan[4], Shoji Torii[28], Harufumi Tsuchiya[29], Shigeharu Udo[12], Shuo Wang[10], Shifeng Wang[6], Yaping Wang[6], Wangdui[6], Qi Wu[6], Liang Xue[9], Jia Yin[5], Yoshichika Yokoe[2], Yanlin Yu[4,14], Aifang Yuan[6], Liuming Zhai[5], Jilong Zhang[4], Xiao Zhang[3], Xueyao Zhang[9], Ying Zhang[4] and Yihuan Zou[4,14]

[1]Department of Physics, Hirosaki University, Hirosaki 036-8561, Japan.
[2]Institute for Cosmic Ray Research, University of Tokyo, Kashiwa 277-8582, Japan.
[3]School of Astronomy and Space Science, Nanjing University, Nanjing 210093, China.
[4]State Key Laboratory of Particle Astrophysics, Institute of High Energy Physics, Chinese Academy of Sciences, Beijing 100049, China.
[5]National Astronomical Observatories, Chinese Academy of Sciences, Beijing 100101, China.
[6]The Key Laboratory of Cosmic Rays (Tibet University), Ministry of Education, Lhasa 850000, China.
[7]Department of Physics, Hebei Normal University, Shijiazhuang 050016, China.
[8]Research Center for Astronomical Computing, Zhejiang Laboratory, Hangzhou 311121, China.
[9]Institute of Frontier and Interdisciplinary Science and Key Laboratory of Particle Physics and Particle Irradiation (MOE), Shandong University, Qingdao 266237, China.
[10]Shandong Key Laboratory of Optical Astronomy and Solar-Terrestrial Environment, School of Space Science and Physics, Institute of Space Sciences, Shandong University, Weihai 264209, China.
[11]Department of Physics, Shinshu University, Matsumoto 390-8621, Japan.
[12]Faculty of Engineering, Kanagawa University, Yokohama 221-8686, Japan.
[13]Faculty of Education, Utsunomiya University, Utsunomiya 321-8505, Japan.
[14]University of Chinese Academy of Sciences, Beijing 100049, China.
[15]Institute of Modern Physics, SouthWest Jiaotong University, Chengdu 610031, China.
[16]Faculty of Systems Engineering, Shibaura Institute of Technology, Omiya 330-8570, Japan.
[17]Faculty of Engineering, Yokohama National University, Yokohama 240-8501, Japan.
[18]Institut d'Astrophysique de Paris, CNRS UMR 7095, Sorbonne Université, 98 bis bd Arago 75014, Paris, France.
[19]Polar Environment Data Science Center, Joint Support-Center for Data Science Research, Research Organization of Information and Systems, Tachikawa 190-0014, Japan.
[20]National Center for Space Weather, China Meteorological Administration, Beijing 100081, China.
[21]School of Information and Intelligent Transportation, Fujian Chuanzheng Communications College, Fuzhou 350007, China.
[22]National Institute of Informatics, Tokyo 101-8430, Japan.
[23]School of Intelligent Engineering, Shandong Management University, Jinan 250357, China.
[24]College of Science, China University of Petroleum, Qingdao 266555, China.
[25]Tokyo Metropolitan College of Industrial Technology, Tokyo 116-8523, Japan.
[26]Department of Information and Electronics, Nagano Prefectural Institute of Technology, Ueda 386-1211, Japan.
[27]College of Industrial Technology, Nihon University, Narashino 275-8575, Japan.
[28]Research Institute for Science and Engineering, Waseda University, Tokyo 162-0044, Japan.
[29]Japan Atomic Energy Agency, Tokai-mura 319-1195, Japan.

SUPPLEMENTARY TEXT

PROPAGATION OF ELECTRONS AND POSITRONS

The diffusion-loss equation describes the electron/positron propagation in the ISM as,

$$\frac{\partial N(E,\boldsymbol{r},t)}{\partial t} - D\Delta N(E,\boldsymbol{r},t) + \frac{\partial[b(E)N(E,\boldsymbol{r},t)]}{\partial E} = Q(E,\boldsymbol{r},t)\,, \tag{1}$$

where N is the differential number density of electrons/positrons with an energy E at a location $\boldsymbol{r}$ and time t, D is the spatial diffusion coefficient, and $b = dE/dt$ is the energy-loss rate of electrons/positrons. The second and third terms on the left-hand side of this equation represent the diffusion and radiative energy loss, respectively, while $Q(E,\boldsymbol{r},t)$ on the right-hand side is the source function determined by injection rate of electrons/positrons from PWN into the ISM. For TeV electrons/positrons, the energy loss is dominated by the synchrotron radiation and inverse Compton scattering (ICS). We adopt the formula in Ref.(*37*) for the energy-loss rate, in which the Klein-Nishina effect of the ICS is accurately taken into account. The magnetic field magnitude of 3 μG is assumed to calculate the synchrotron loss. The target photon field needed to calculate the ICS is taken from Ref.(*38*).

As the scale of Geminga PWN is significantly smaller than the TeV halo, we can safely assume a point source. We use the spin-down profile of the pulsar luminosity for the time dependence of Q. Thus, the source term is written, as

$$Q(E,\boldsymbol{r},t) = q(E)\,\delta(\boldsymbol{r}-\boldsymbol{r}_s)\,(1+\frac{t}{\tau_0})^{-2}\,, \tag{2}$$

where $q(E)$ is the injection spectrum of electrons/positrons, $\boldsymbol{r}_s$ is the source location, τ_0 is the spin-down time scale of pulsar which we set $\tau_0 = 10$ kyr, and δ is the Dirac function. As the cooling time of 100 TeV electrons is around 10 kyr and much shorter than the Geminga's age of 340 kyr, the parent electrons/positrons of TeV γ-ray halo are generated at a very late age of Geminga.

The electron energy is converted from the spin-down energy of pulsar with an efficiency of η:

$$\int_{E_1}^{E_2} q(E)E(1+\frac{t}{\tau_0})^{-2}dE = \eta L(t)\,, \tag{3}$$

where $L(t) \propto (1+t/\tau_0)^{-2}$ is the spin-down luminosity of pulsar. By using the magnitude of current $L(t)$ at $t = 340$ kyr known to be 3.25×10^{34} erg s^{-1}, we can calculate $L(t)$ at any time. As seen in Eq. (3), the injection spectrum of electrons/positrons is normalized to $L(t)$ by η for a given spectral shape. The minimum and maximum energy (E_1 and E_2 in Eq. (3)) are set at 1 GeV and 500 TeV, respectively.

Eq. (1) is solved with the Green function method, assuming a 3D infinite boundary condition, as the propagation scale of high-energy electrons/positrons around Geminga is negligible compared with the Galactic diffusion zone scale. We can calculate the γ-ray surface brightness and its energy spectrum once we determine the present electron/positron number density $N(E,\boldsymbol{r},t)$.

DETERMINATION OF THE ELECTRON/POSITRON SPECTRUM

The electron/positron injection spectrum $q(E)$ in the source term of Eq. (2) reflects the acceleration power of the PWN. The energy spectrum of halo γ-rays is mainly determined by $q(E)$, while it weakly depends on the diffusion coefficient $D(E)$. Conversely, the γ-ray surface brightness profile (SBP), which characterizes the spatial distribution of electrons, is predominantly governed by the diffusion coefficient $D(E)$.

In our calculation, the observed halo γ-ray spectrum is integrated over a circular window with an angular radius of 3° around the PWN, suppress the contamination from the background events. The best-fit parameters for both PL and ECPL models are given in the main text. The fitted γ-ray SBP provides the ratio of γ-ray flux within 3° to the total flux. We obtain the total γ-ray spectrum by multiplying the observed flux by this factor.

As the energy dependence of diffusion coefficient cannot be well constrained in this step, we have assumed in the main text $\delta = 1/3$, which is expected from the Komolgorov turbulence. We also examined the case of $\delta = 1/2$, e.g., the Kraichnan type of turbulence, and obtained $E_c = 104^{+27}_{-21}$ TeV and $\eta = 0.110^{+0.037}_{-0.028}$ for ECPL model being consistent with the result of the $\delta = 1/3$ case. Therefore, the assumption of $\delta = 1/3$ results in a minor influence on the obtained injection spectrum $q(E)$.

Determination of diffusion coefficient in different energy bins

We derive the γ-ray SBP from the calculated electron/positron density distribution. By fitting to the SBP in different energy bins, the diffusion coefficient $D(E)$ is determined. We determine the SBPs from the best-fit D(E) and show them in Figs. 2B in the main text.

In the following, we describe the relation between the energy of γ-ray SBP in each energy bin and the corresponding electron/positron energy E which determines $D(E)$.

We denote by $f_1(\epsilon, E)$ the spectrum of photons with energy ϵ produced by a single electron with energy E. Then, the energy distribution of the parent electrons is related to f_1, as

$$f(\epsilon, E) = \int f_1(\epsilon, E) N(E, \boldsymbol{r})\, dV = \int_0^{10^\circ} \int_\theta f_1(\epsilon, E) N(E, \boldsymbol{r}) dl_\theta\, 2\pi\theta d\theta \tag{4}$$

where $N(E, \boldsymbol{r})$ is the electron spectrum at present. We first integrate along the line of sight (l_θ) at angular distance θ from the PWN and then integrate over θ up to 10° where the Tibet ASγ measures no halo γ-rays anymore. By using f calculated in Eq. (3), we obtain the weighted average energy of an electron/positron, as

$$\bar{E}(\epsilon) = \frac{\int f(\epsilon, E) E dE}{\int f(\epsilon, E) dE}\ . \tag{5}$$

For ϵ in each energy bin, we use the central energy in the bin.

FITTING RESULTS TO THE γ-RAY HALO SPECTRUM

Fig. S1 shows the fitting results in case of the power-law spectrum assumed for injection electrons/positrons. The maximum likelihood and posterior probability distribution of the parameters obtained with the Bayesian inference tool MULTINEST (*39*) are shown. It is seen that the efficiency parameter $\log\eta$ has a strong correlation with the power-law index α of γ-ray spectrum. A soft spectrum with large α results in a large η. It is found that $\eta < 1$ is excluded at 3σ C.L.. This means that the single power-law model of electrons/positrons injection spectrum is significantly disfavored.

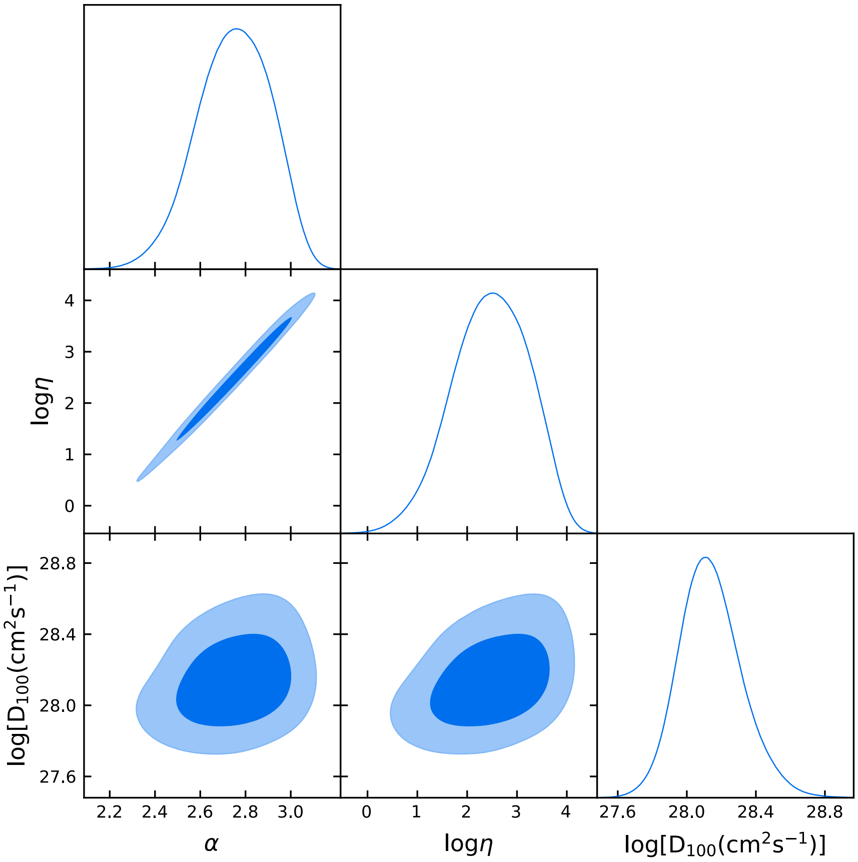

FIG. S1: **1D and 2D posterior probability distributions of the model parameters in the power-law injection electrons/positrons scenario.** The dark and light blue shaded areas are the 68% and 95% confidence intervals, respectively. The plot is drawn with GetDist (*40*).

REFERENCES

1. P. Blasi, The origin of galactic cosmic rays. *Astron. Astrophys. Rev.* **21**, 70 (2013).

2. T. K. Gaisser, *Cosmic Rays and Particle Physics* (Cambridge Univ. Press, 1990).

3. V. L. Ginzburg, Cosmic rays and plasma phenomena in the Galaxy and Metagalaxy. *Sov. Astron.* **9**, 877 (1966).

4. V. S. Berezinsky, S. V. Bulanov, V. A. Dogiel, V. L. Ginzburg, V. S. Ptuskin, *Astrophysics of Cosmic Rays* (North-Holland, 1990).

5. O. Adriani, G. C. Barbarino, G. A. Bazilevskaya, R. Bellotti, M. Boezio, E. A. Bogomolov, L. Bonechi, M. Bongi, V. Bonvicini, S. Borisov, S. Bottai, A. Bruno, F. Cafagna, D. Campana, R. Carbone, P. Carlson, M. Casolino, G. Castellini, L. Consiglio, M. P. De Pascale, C. De Santis, N. De Simone, V. Di Felice, A. M. Galper, W. Gillard, L. Grishantseva, P. Hofverberg, G. Jerse, A. V. Karelin, S. V. Koldashov, S. Y. Krutkov, A. N. Kvashnin, A. Leonov, V. Malvezzi, L. Marcelli, A. G. Mayorov, W. Menn, V. V. Mikhailov, E. Mocchiutti, A. Monaco, N. Mori, N. Nikonov, G. Osteria, P. Papini, M. Pearce, P. Picozza, C. Pizzolotto, M. Ricci, S. B. Ricciarini, L. Rossetto, M. Simon, R. Sparvoli, P. Spillantini, Y. I. Stozhkov, A. Vacchi, E. Vannuccini, G. Vasilyev, S. A. Voronov, J. Wu, Y. T. Yurkin, G. Zampa, N. Zampa, V. G. Zverev, PAMELA results on the cosmic-ray antiproton flux from 60 MeV to 180 GeV in kinetic energy. *Phys. Rev. Lett.* **105**, 121101 (2010).

6. M. Aguilar, L. A. Cavasonza, B. Alpat, G. Ambrosi, L. Arruda, N. Attig, S. Aupetit, P. Azzarello, AMS Collaboration, Antiproton flux, antiproton-to-proton flux ratio, and properties of elementary particle fluxes in primary cosmic rays measured with the Alpha Magnetic Spectrometer on the International Space Station. *Phys. Rev. Lett.* **117**, 091103 (2016).

7. O. Adriani, G. C. Barbarino, G. A. Bazilevskaya, R. Bellotti, M. Boezio, E. A. Bogomolov, L. Bonechi, M. Bongi, V. Bonvicini, S. Bottai, A. Bruno, F. Cafagna, D. Campana, R. Carbone, M. Casolino, G. Castellini, M. P. De Pascale, G. De Rosa, C. De Santis, N. De Simone, V. Di Felice, A. M. Galper, L. Grishantseva, P. Hofverberg, S. V. Koldashov, S. Y. Krutkov, A. N. Kvashnin, A. Leonov, V. Malvezzi, L. Marcelli, W. Menn, V. V. Mikhailov, E. Mocchiutti, S. Orsi, G. Osteria, P. Papini, M. Pearce, P. Picozza, M. Ricci, S. B. Ricciarini, M. Simon, R.

Sparvoli, P. Spillantini, Y. I. Stozhkov, A. Vacchi, E. Vannuccini, G. Vasilyev, S. A. Voronov, Y. T. Yurkin, G. Zampa, N. Zampa, V. G. Zverev, An anomalous positron abundance in cosmic rays with energies 1.5–100 GeV. *Nature* **458**, 607–609 (2009).

8. L. Accardo, M. Aguilar, D. Aisa, B. Alpat, A. Alvino, G. Ambrosi, K. Andeen, L. Arruda, AMS Collaboration, High statistics measurement of the positron fraction in primary cosmic rays of 0.5-500 GeV with the alpha magnetic spectrometer on the international space station. *Phys. Rev. Lett.* **113**, 121101 (2014).

9. D. Hooper, P. Blasi, P. D. Serpico, Pulsars as the sources of high energy cosmic ray positrons. *J. Cosmol. Astropart. Phys.* **2009**, 025 (2009).

10. P. F. Yin, Z. H. Yu, Q. Yuan, X. J. Bi, Pulsar interpretation for the AMS-02 result. *Phys. Rev. D* **88**, 023001 (2013).

11. S. Profumo, Dissecting cosmic-ray electron-positron data with Occam's Razor: The role of known Pulsars. *Central Eur. J. Phys.* **10**, 1–31 (2012).

12. B. M. Gaensler, P. O. Slane, The evolution and structure of pulsar wind nebulae. *Ann. Rev. Astron. Astrophys.* **44**, 17–47 (2006).

13. A. U. Abeysekara, A. Albert, R. Alfaro, C. Alvarez, J. D. Álvarez, R. Arceo, J. C. Arteaga-Velázquez, D. Avila Rojas, H. A. Ayala Solares, A. S. Barber, N. Bautista-Elivar, A. Becerril, E. Belmont-Moreno, S. Y. BenZvi, D. Berley, A. Bernal, J. Braun, C. Brisbois, K. S. Caballero-Mora, T. Capistrán, A. Carramiñana, S. Casanova, M. Castillo, U. Cotti, J. Cotzomi, S. Coutiño de León, C. De León, E. De la Fuente, B. L. Dingus, M. A. DuVernois, J. C. Díaz-Vélez, R. W. Ellsworth, K. Engel, O. Enríquez-Rivera, D. W. Fiorino, N. Fraija, J. A. García-González, F. Garfias, M. Gerhardt, A. González Muñoz, M. M. González, J. A. Goodman, Z. Hampel-Arias, J. P. Harding, S. Hernández, A. Hernández-Almada, J. Hinton, B. Hona, C. M. Hui, P. Hüntemeyer, A. Iriarte, A. Jardin-Blicq, V. Joshi, S. Kaufmann, D. Kieda, A. Lara, R. J. Lauer, W. H. Lee, D. Lennarz, H. León Vargas, J. T. Linnemann, A. L. Longinotti, G. Luis Raya, R. Luna-García, R. López-Coto, K. Malone, S. S. Marinelli, O. Martinez, I. Martinez-Castellanos, J. Martínez-Castro, H. Martínez-Huerta, J. A. Matthews, P. Miranda-Romagnoli, E. Moreno, M. Mostafá, L. Nellen, M. Newbold, M. U. Nisa, R.

Noriega-Papaqui, R. Pelayo, J. Pretz, E. G. Pérez-Pérez, Z. Ren, C. D. Rho, C. Rivière, D. Rosa-González, M. Rosenberg, E. Ruiz-Velasco, H. Salazar, F. Salesa Greus, A. Sandoval, M. Schneider, H. Schoorlemmer, G. Sinnis, A. J. Smith, R. W. Springer, P. Surajbali, I. Taboada, O. Tibolla, K. Tollefson, I. Torres, T. N. Ukwatta, G. Vianello, T. Weisgarber, S. Westerhoff, I. G. Wisher, J. Wood, T. Yapici, G. Yodh, P. W. Younk, A. Zepeda, H. Zhou, F. Guo, J. Hahn, H. Li, H. Zhang, Extended gamma-ray sources around pulsars constrain the origin of the positron flux at Earth. *Science* **358**, 911 (2017).

14. A. M. W. Mitchell, S. Caroff, J. Hintonc, L. Mohrmannd on behalf of the H.E.S.S. Collaboration, Detection of extended TeV emission around the Geminga pulsar with H.E.S.S. arXiv:2108.02556 [astro-ph.HE] (2022).

15. A. Albert, R. Alfaro, C. Alvarez, J. C. Arteaga-Velázquez, D. Avila Rojas, H. A. Ayala Solares, R. Babu, E. Belmont-Moreno, A. Bernal, K. S. Caballero-Mora, T. Capistrán, A. Carramiñana, S. Casanova, U. Cotti, J. Cotzomi, S. Coutiño de León, E. de la Fuente, D. Depaoli, N. Di Lalla, R. Diaz Hernandez, B. L. Dingus, M. A. DuVernois, M. Durocher, J. C. Díaz-Vélez, K. Engel, C. Espinoza, K. L. Fan, K. Fang, N. Fraija, J. A. García-González, F. Garfias, H. Goksu, M. M. González, J. A. Goodman, S. Groetsch, J. P. Harding, S. Hernández-Cadena, I. Herzog, P. Hüntemeyer, D. Huang, F. Hueyotl-Zahuantitla, A. Iriarte, V. Joshi, S. Kaufmann, D. Kieda, A. Lara, W. H. Lee, J. Lee, H. León Vargas, J. T. Linnemann, A. L. Longinotti, G. Luis-Raya, K. Malone, O. Martinez, J. Martínez-Castro, J. A. Matthews, P. Miranda-Romagnoli, J. A. Montes, J. A. Morales-Soto, E. Moreno, M. Mostafá, A. Nayerhoda, L. Nellen, R. Noriega-Papaqui, L. Olivera-Nieto, N. Omodei, Y. Pérez Araujo, E. G. Pérez-Pérez, C. D. Rho, D. Rosa-González, H. Salazar, D. Salazar-Gallegos, A. Sandoval, M. Schneider, G. Schwefer, J. Serna-Franco, Y. Son, R. W. Springer, O. Tibolla, K. Tollefson, I. Torres, R. Torres-Escobedo, R. Turner, F. Urea-Mena, E. Varela, L. Villaseñor, X. Wang, I. J. Watson, E. Willox, H. Wu, S. Yun-Cárcamo, H. Zhou, C. de León, M. Di Mauro, Precise measurements of TeV halos around Geminga and Monogem pulsars with HAWC. *Astrophys. J.* **974**, 246–258 (2024).

16. M. Aguilar, L. A. Cavasonza, G. Ambrosi, L. Arruda, N. Attig, S. Aupetit, P. Azzarello, A. Bachlechner, AMS Collaboration, Precision measurement of the boron to carbon flux ratio in

cosmic rays from 1.9 GV to 2.6 TV with the Alpha Magnetic Spectrometer on the International Space Station. *Phys. Rev. Lett.* **117**, 231102 (2016).

17. A. Kolmogorov, The local structure of turbulence in incompressible viscous fluid for very large Reynolds numbers. *Dokl. Akad. Nauk SSSR* **30**, 301–305 (1941).

18. J. Faherty, F. M. Walter, J. Anderson, The trigonometric parallax of the neutron star Geminga. *Astrophys. Space Sci.* **308**, 225–230 (2007).

19. G. G. Pavlov, S. Bhattacharyya, V. E. Zavlin, New x-ray observations of the Geminga pulsar wind nebula. *Astrophys. J.* **715**, 66–77 (2010).

20. B. Posselt, G. G. Pavlov, P. O. Slane, R. Romani, N. Bucciantini, A. M. Bykov, O. Kargaltsev, M. C. Weisskopf, C. Y. Ng, Geminga's puzzling pulsar wind nebula. *Astrophys. J.* **835**, 66 (2017).

21. P. A. Caraveo, G. F. Bignami, A. DeLuca, S. Mereghetti, A. Pellizzoni, R. Mignani, A. Tur, W. Becker, Geminga's tails: A pulsar bow shock probing the interstellar medium. *Science* **301**, 1345–1347 (2003).

22. S.-Q. Xi, R.-Y. Liu, Z.-Q. Huang, K. Fang, X.-Y. Wang, GeV observations of the extended pulsar wind nebulae constrain the pulsar interpretations of the cosmic-ray positron excess. *Astrophys. J.* **878**, 104 (2019).

23. A. A. Abdo, B. T. Allen, T. Aune, D. Berley, C. Chen, G. E. Christopher, T. DeYoung, B. L. Dingus, R. W. Ellsworth, M. M. Gonzalez, J. A. Goodman, E. Hays, C. M. Hoffman, P. H. Huntemeyer, B. E. Kolterman, J. T. Linnemann, J. E. McEnery, T. Morgan, A. I. Mincer, P. Nemethy, J. Pretz, J. M. Ryan, P. M. S. Parkinson, A. Shoup, G. Sinnis, A. J. Smith, V. Vasileiou, G. P. Walker, D. A. Williams, G. B. Yodh, Milagro observations of multi-TeV emission from galactic sources in the fermi bright source list. *Astrophys. J. Lett.* **700**, L127–L131 (2009).

24. J. L. Han, K. Ferriere, R. N. Manchester, The spatial energy spectrum of magnetic fields in our galaxy. *Astrophys. J.* **610**, 820–826 (2004).

25. A. H. Minter, S. R. Spangler, Observation of turbulent fluctuations in the interstellar plasma density and magnetic field on spatial scales of 0.01 to 100 parsecs. *Astrophys. J.* **458**, 194 (1996).

26. C. Evoli, T. Linden, G. Morlino, Self-generated cosmic-ray confinement in TeV halos: Implications for TeV γ-ray emission and the positron excess. *Phys. Rev. D* **98**, 063017 (2018).

27. P. Mukhopadhyay, T. Linden, Self-generated cosmic-ray turbulence can explain the morphology of TeV halos. *Phys. Rev. D* **105**, 123008 (2022).

28. K. Fang, X.-J. Bi, P.-F. Yin, Possible origin of the slow-diffusion region around Geminga. *Mon. Not. R. Astron. Soc.* **488**, 4074–4080 (2019).

29. M. Amenomori, Y. W. Bao, X. J. Bi, D. Chen, T. L. Chen, W. Y. Chen, X. Chen, Y. Chen, Tibet ASγ Collaboration, First detection of photons with energy beyond 100 TeV from an astrophysical source. *Phys. Rev. Lett.* **123**, 051101 (2019).

30. T. K. Sako, K. Kawata, M. Ohnishi, A. Shiomi, M. Takita, H. Tsuchiya, Exploration of a 100 TeV gamma-ray northern sky using the Tibet air-shower array combined with an underground water-Cherenkov muon-detector array. *Astropart. Phys.* **32**, 177–184 (2009).

31. K. Kawata, T. K. Sako, M. Ohnishi, M. Takita, Y. Nakamura, K. Munakata, Energy determination of gamma-ray induced air showers observed by an extensive air shower array. *Exp. Astron.* **44**, 1–9 (2017).

32. M. Amenomori, X. J. Bi, D. Chen, S. W. Cui, Danzengluobu, L. K. Ding, X. H. Ding, C. Fan, C. F. Feng, Z. Feng, Z. Y. Feng, X. Y. Gao, Q. X. Geng, H. W. Guo, H. H. He, M. He, K. Hibino, N. Hotta, H. Hu, H. B. Hu, J. Huang, Q. Huang, H. Y. Jia, F. Kajino, K. Kasahara, Y. Katayose, C. Kato, K. Kawata, Labaciren, G. M. Le, A. F. Li, J. Y. Li, Y.-Q. Lou, H. Lu, S. L. Lu, X. R. Meng, K. Mizutani, J. Mu, K. Munakata, A. Nagai, H. Nanjo, M. Nishizawa, M. Ohnishi, I. Ohta, H. Onuma, T. Ouchi, S. Ozawa, J. R. Ren, T. Saito, T. Y. Saito, M. Sakata, T. K. Sako, M. Shibata, A. Shiomi, T. Shirai, H. Sugimoto, M. Takita, Y. H. Tan, N. Tateyama, S. Torii, H. Tsuchiya, S. Udo, B. Wang, H. Wang, X. Wang, Y. Wang, Y. G. Wang, H. R. Wu, L. Xue, Y. Yamamoto, C. T. Yan, X. C. Yang, S. Yasue, Z. H. Ye, G. C. Yu, A. F.

Yuan, T. Yuda, H. M. Zhang, J. L. Zhang, N. J. Zhang, X. Y. Zhang, Y. Zhang, Y. Zhang, Zhaxisangzhu, X. X. Zhou, Multi-TeV gamma-ray observation from the crab nebula using the Tibet-III air shower array finely tuned by the cosmic ray Moon's shadow. *Astrophys. J.* **692**, 61–72 (2009).

33. A. A. Abdo, B. Allen, T. Aune, D. Berley, E. Blaufuss, S. Casanova, C. Chen, B. L. Dingus, R. W. Ellsworth, L. Fleysher, R. Fleysher, M. M. Gonzalez, J. A. Goodman, C. M. Hoffman, P. H. Huntemeyer, B. E. Kolterman, C. P. Lansdell, J. T. Linnemann, J. E. McEnery, A. I. Mincer, P. Nemethy, D. Noyes, J. Pretz, J. M. Ryan, P. M. Saz Parkinson, A. Shoup, G. Sinnis, A. J. Smith, G. W. Sullivan, V. Vasileiou, G. P. Walker, D. A. Williams, G. B. Yodh, Discovery of localized regions of excess 10-TeV cosmic rays. *Phys. Rev. Lett.* **101**, 221101 (2008).

34. M. Amenomori, S. Ayabe, S. W. Cui, Danzengluobu, L. K. Ding, X. H. Ding, C. F. Feng, Z. Y. Feng, X. Y. Gao, Q. X. Geng, H. W. Guo, H. H. He, M. He, K. Hibino, N. Hotta, H. Hu, H. B. Hu, J. Huang, Q. Huang, H. Y. Jia, F. Kajino, K. Kasahara, Y. Katayose, K. Kawata, Labaciren, G. M. Le, J. Y. Li, H. Lu, S. L. Lu, X. R. Meng, K. Mizutani, J. Mu, H. Nanjo, M. Nishizawa, M. Ohnishi, I. Ohta, T. Ouchi, S. Ozawa, J. R. Ren, T. Saito, M. Sakata, T. Sasaki, M. Shibata, A. Shiomi, T. Shirai, H. Sugimoto, K. Taira, M. Takita, Y. H. Tan, N. Tateyama, S. Torii, H. Tsuchiya, S. Udo, T. Utsugi, B. S. Wang, H. Wang, X. Wang, Y. G. Wang, L. Xue, Y. Yamamoto, X. C. Yang, Z. H. Ye, G. C. Yu, A. F. Yuan, T. Yuda, H. M. Zhang, J. L. Zhang, N. J. Zhang, X. Y. Zhang, Y. Zhang, Zhaxisangzhu, X. X. Zhou, Multi-tev gamma-ray flares from markarian 421 in 2000 and 2001 observed with the Tibet air shower array. *Astrophys. J.* **598**, 242–249 (2003).

35. G. R. Blumenthal, R. J. Gould, Bremsstrahlung, synchrotron radiation, and Compton scattering of high-energy electrons traversing dilute gases. *Rev. Mod. Phys.* **42**, 237–270 (1970).

36. Q. Yuan, S.-J. Lin, K. Fang, X.-J. Bi, Propagation of cosmic rays in the AMS-02 era. *Phys. Rev. D* **95**, 083007 (2017).

37. K. Fang, X. J. Bi, S. J. Lin, Q. Yuan, Klein-Nishina effect and the cosmic ray electron spectrum. *Chin. Phys. Lett.* **38**, 039801 (2021).

38. T. Delahaye, J. Lavalle, R. Lineros, F. Donato, N. Fornengo, Galactic electrons and positrons at the Earth: New estimate of the primary and secondary fluxes. *Astron. Astrophys.* **524**, A51 (2010).

39. F. Feroz, M. P. Hobson, M. Bridges, MultiNest: An efficient and robust Bayesian inference tool for cosmology and particle physics. *Mon. Not. R. Astron. Soc.* **398**, 1601–1614 (2009).

40. A. Lewis, GetDist: A Python package for analysing Monte Carlo samples. arXiv:1910.13970 [astro-ph.IM] (2019).
